# Making a Diagnosis of Common Variable Immunodeficiency: A Review

**DOI:** 10.7759/cureus.6711

**Published:** 2020-01-20

**Authors:** Asma Ghafoor, Shona M Joseph

**Affiliations:** 1 Allergy and Immunology, Nova Southeastern University School of Osteopathic Medicine, Davie, USA

**Keywords:** common variable immunodeficiency, cvid, immunoglobulins, recurrent sinopulmonary infections

## Abstract

Common variable immunodeficiency (CVID) is a condition that inhibits the function of the immune system, making those with the condition more susceptible to infection from external pathogens, including bacteria and, less often, viruses. The immune disorder is marked by low immunoglobulin levels of immunoglobulin G (IgG) and IgA as well as IgM in some patients. These immune abnormalities typically result in recurrent sinopulmonary infections and can result in serious complications such as pneumonia and chronic lung disease. Other manifestations include poor vaccine response and defective antibodies in a patient’s immune system. The disorder affects approximately one in 25,000 to one in 50,000 individuals worldwide, with the condition varying across different populations. While the underlying mechanism of disease activity remains poorly understood, only 10% of cases are known to have an underlying genetic link and approximately 25% of patients also have an autoimmune disorder. CVID commonly presents in individuals in their twenties or thirties but can present at any time between childhood through adulthood, with mortality dependent on the severity of illness and frequency of recurrent infections. Potential life-threatening consequences of CVID include malignancies, enteropathy, and autoimmune manifestations. Treatment can help alleviate symptoms and prevent continued recurrent infections and serious complications. However, the lack of awareness among primary care physicians (PCPs) makes the condition difficult to diagnose and manage. In this review article, we will provide insight into the clinical manifestations as well as the diagnosis and management of CVID. This will provide clinical practitioners with tools to recognize the disease earlier on to improve patient outcomes and prevent serious complications. We will also afford a better understanding of genetic components tied to CVID and new research efforts.

## Introduction and background

Common variable immunodeficiency (CVID) is an autoimmune disorder that is associated with recurrent infections and low antibody levels, where B cells fail to differentiate, leading to a deficiency of immunoglobulins, specifically immunoglobulin G (IgG) and IgA, and sometimes IgM [1-2]. The condition affects approximately one in every 25,000 individuals to one in every 50,000 individuals, with the disorder varying across different populations and regions [3]. Most individuals with CVID present in their twenties and thirties but the condition can affect children, adolescents, and older adults [3-5]. Diagnosis remains difficult because the condition mimics other immune conditions and a lack of awareness exists among practicing clinicians [6]. Due to insufficient awareness and many individuals with CVID going undiagnosed, infections may continue to recur, which can lead to more serious co-morbid conditions. Therefore, building awareness among primary care physicians (PCPs) is very important, as treatment can help alleviate symptoms and prevent continued recurrent infections and serious complications [7]. This review article will provide insight into CVID's clinical manifestations, diagnosis, physiology/molecular basis, treatment, genetics, effects of recurrent infections, diagnostic tools, and case studies.

## Review

Physiology/molecular basis

B lymphocytes (B-cells), a type of white blood cell (WBC) derived from bone marrow in the adaptive immune system (the sub-system of the overall immune system that eliminates pathogens and/or prevents their growth), primarily known for secreting antibodies. B-cells can differentiate into two types of cells, plasma cells or memory B-cells, or they can be secreted as immunoglobulins IgG, IgA, and IgM. CVID patients present with a decrease in IgG [8]. Low levels of IgA and IgM can also be present [8]. Still, the exact mechanism of action remains unknown, but researchers have demonstrated irregular gene rearrangement, decreased naïve B-cell stock, complications that occur in the bone marrow as B-cells are mature, and impairment in b-cell binding to toll-like receptors that may result in the abnormal activation of antibody secretion [2]. Furthermore, there is also a correlation between low T lymphocyte (T-cells) levels and CVID [2]. T-cells are a type of WBC in the adaptive immune system, which mature in the thymus and differentiate into cells involved in lymphocyte maturation or are involved in killing cells infected by virus via cell-mediated immunity. Studies have shown a connection between CVID and T-cell proliferation and subsequent cytokine release.

Genetics

The majority of CVID cases arise randomly and are not associated with genetic susceptibility. The condition is characterized by low immunoglobulin levels that cause dysfunctions in B-cells and T-cells [5]. Still, approximately, between 5% and 25% of cases are related to familial inheritance. CVID is most commonly tied to a monogenic mode of inheritance, with either recessive or dominant allele mutations, but there is a small fraction of patients with polygenic or epigenetic modes of inheritance that can be influenced by environmental triggers [4]. DNA sequencing techniques have been used to diagnose the genetic causes of CVID but these can be expensive and time-consuming [8]. Whole exome sequencing (WES) could be used to identify defects in particular sequences coding for proteins in CVID patients [8]. Such genetic defects account for approximately 85% of disease-related sequences, yet only account for 1% of the entire genome [8]. In a study conducted by Jørgensen et al., the researchers proposed that gut microbiota and gastrointestinal (GI) inflammation have a role in the systemic activation of CVID [5]. This variability in incidence in the population of CVID patients suggests that there may be an environmental component [5]. Current research has not identified any specific environmental triggers, but limited research has been completed to date [4-6].

Clinical manifestations

Most individuals with CVID present in their twenties and thirties but the condition can affect children, adolescents, and older adults [3]. However, CVID can arise in patients in various stages of life. Some CVID patients feel symptoms as early as the first decade of life, but patients can present as late as 70 years of age [5]. CVID can present with impacts on different organ systems, but most commonly, it can negatively affect the gastrointestinal and lymphatic systems [4]. Abdominal pain, nausea, vomiting, and weight loss are some of the most common symptoms patients typically present to physicians with [3]. Enlarged lymph nodes and splenomegaly may also occur as a result of recurrent infections, most commonly lung infections [3]. Some patients can have sequelae like joint inflammation, arthritis, anemia, and endocrine disorders as a result of the production of autoantibodies [9].

A potential late clinical manifestation that may be overlooked is the increased risk of gastric cancers and lymphomas [4]. Studies have shown an increased risk of gastric cancers in patients with CVID [4]. It is believed that this is a result of Helicobacter pylori (HP) infections [4]. Antibodies have, however, eradicated most of these infections [4]. It is more widely accepted now that the mechanism of action is the decreased production of gastric IgA and hydrochloric acid, which causes HP infections to persist [4]. HP infections also cause the upregulation of oncogenes and the downregulation of tumor suppressor genes and increase nitrosation, which can lead to the progression of metaplasia to dysplasia and, eventually, oncologic changes [4]. Other effects like inflammation can trigger mutations that alter genes involved in cell replication and death, which can also lead to carcinoma. As for gastric lymphomas, gastrointestinal (GI) infections have been linked to CVID-associated lymphoproliferation [4]. While the symptoms of CVID may overlap with other disorders, it is important to not rule it out immediately, especially due to the increased risk of cancer.

Diagnosis

Currently, no standardized criteria for the diagnosis of CVID have been established and physician awareness remains low [1,3]. Diagnosis remains difficult because of this low awareness among physicians, and the condition has been shown to mimic other immune conditions. CVID is a diagnosis of exclusion and, therefore, requires specific testing to hone in on a definitive diagnosis [6]. One definitive characteristic of CVID is the presence of hypogammaglobulinemia with reduced IgA and, sometimes, concomitant IgM deficiency [1]. CVID patients, in addition to reduced IgA and IgM levels, may also have poor vaccine response and frequent sinopulmonary infections (which is also common among those who are only IgG deficient) [1]. In the study conducted by Filion et al., patients with CVID and those that are IgG isotype deficient were compared clinically and immunologically [1]. Both subgroups of patients possessed B-cell defects along with some degree of decreased antibody levels [1]. Due to the reduced levels of IgA and IgM, in addition to IgG, patients with CVID had greater B-cell defects than those who are IgG isotype deficient alone [1]. The CVID patients were also noted to have less response to vaccinations, specifically to protein and polysaccharide vaccines, and a greater probability of autoimmune cytopenias, interstitial lung disease, splenomegaly, and other health issues [1]. In addition, CVID patients do not possess fully functioning antibodies, leading to this increased incidence of infections [1]. All secondary causes that may cause low levels of IgG must be excluded before a final diagnosis of CVID is reached [1]. Furthermore, patients with large T-cell deficiencies must also be ruled out before making the diagnosis [5]. CVID is associated with some platelet disorders, such as granulocytopenia, lymphocytopenia, small platelets, and thrombocytopenia, so a complete blood count (CBC) with differential would be helpful. Immunoglobulin levels can also rule in or out other diseases, such as hyper IgM syndrome. B-cell memory, and naive panels, can also be used to include and/or exclude other disease processes. CVID will display low levels of memory B-cells [6]. B-cell and CD4 T-cell titers should also be measured, as CVID has been shown to be associated with low CD4 T-cell counts [1]. Still, if there is a significant decrease in these levels it might suggest other disease processes [1]. Measuring immunoglobulin levels before and after vaccinations is another diagnostic tool, as noted above, impaired vaccine response can also suggest CVID [1]. Other tests that can rule out differential diagnoses include monoclonal protein levels for monoclonal gammopathy and ferritin levels for hereditary hemochromatosis. In some patients, CVID can manifest with granulomas in the lungs, lymph nodes, liver, and other organs [10]. This can progress to granulomatous lymphocytic interstitial lung disease (GLILD), which can be difficult to detect [10]. In a study by Jolles et al., fluorodeoxyglucose (FDG) positron emission tomography-computed tomography (PET-CT) was shown to significantly aid in detecting and monitoring GLILD in CVID patients [10]. High metabolic activity was detected on the scans, indicating granulomas, and, subsequently, when treated, showed a decrease in metabolic activity [10]. FDG PET-CT scans could potentially be used to monitor for these complications in CVID patients to prevent a worsening of the disease state.

Treatment

Treatment for CVID varies across this patient population, as the condition involves multiple organ systems and relies on symptom and organ system presentation. The drug of choice for CVID and cytopenias are glucocorticoids [11]. High-dose intravenous IG (IVIg), IgG, and anti-D antibodies can also be used [11]. Patients who have undergone splenectomies or are using immunosuppressants, such as rituximab and mycophenolate mofetil must be monitored routinely due to the higher risk of infections from the immune system being suppressed [11]. For patients with antibody deficiencies, 400-600 mg/kg of immunoglobulin per month must be administered throughout the entirety of their lives through either IV or subcutaneous injections (SCIg) [11]. Choosing between IV and SCIg is dependent on multiple factors such as patient characteristics and patient preference [12]. Neither method is considered superior to the other based on current research studies [12]. Treatment with immunoglobulin therapies has been beneficial to patients, as they have been linked to increased life expectancy and reduction in autoimmunity development [12]. Novel treatment approaches include recombinant hyaluronidase-facilitated subcutaneous immunoglobulin (fSCIg), in which recombinant human hyaluronidase is first administered followed by human SCIg 10% in the same infusion [11]. One benefit of this novel infusion therapy is its ability to infuse larger quantities of up to 600 mL of immunoglobulin in one infusion site [11]. The benefits include the ability for patients to come in for fewer treatments, which results in improvements in quality of life [11]. In a study conducted by Pedini et al., four CVID patients who were treated with fSCIg had improvements in autoimmunity because of increased immune homeostasis [11]. The patients in the study were able to reduce their daily steroid dosage of prednisone while taking the fSCIg [11]. Therefore, these infusions may have a steroid-sparing effect [11]. However, more research is needed to evaluate the effects on a larger sample size and for longer periods of time.

Effects of recurrent infections

CVID patients do not produce antibodies on their own and/or have impaired antibody production. Therefore, these patients have a greater probability of acquiring infections, especially recurrent infections [12]. While patients typically present with GI infections at the onset, recurrent infections typically consist of sinus and lung infections [12]. IVIg infusions are given to these patients to compensate for the lack of antibodies that are not being produced within their own bodies [12].

In a study conducted by Gill et al., researchers investigated whether there was a specific time period after receiving IVIg infusions in which patients were most susceptible to acquiring an infection [12]. Although the data had no statistical significance, a notable pattern for the mean days to infection was observed. A total of 22 patients with CVID and one patient with X-linked agammaglobulinemia (XLA) were assessed [11]. Patients were treated with IVIg monthly [12]. The number of infections identified totaled 63 in the 258 visits [12]. Fifty-six of these infections were new infections in 19 of the 23 patients studied [11]. The other four patients did not have any new infections during the research study [12]. Only three of these reported infections began within 21 days from receiving the IVIg infusion [11]. The remainder of the infections that were reported were greater than 21 days after the IVIg infusion was administered [11]. Sinus infections were the most common among patients, with approximately 49.2% of the infections while 33.3% of the infections were from the upper respiratory tract [12]. The remaining infections were in the eyes, ears, or other areas in the body with 6.35% 4.76%, and 6.35% recorded, respectively [12]. The data had no statistical significance but this may have been influenced by factors such as the low sample size [12]. Because the data did not have statistical significance, no conclusions were made from this study [12]. Despite the lack of statistical significance, the mean days to infection was within the third week at 17.0 days like the study hypothesized [12]. Because approximately 95% of new infections happened within the third week and onwards of post IVIg infusion, physicians may want to have heightened awareness when evaluating their patients for infections around that time period because of the “wear-off” effect (when IgG levels approach its trough, typically around four weeks) of the treatment [12]. This is the first study to investigate the timing of infections; future studies should be conducted with an increased sample size.

Case studies

There has been a wide array of case studies following CVID patients. Two will be discussed below. In one study by Więsik-Szewczyk et al., the patient was a 31-year old female with a history of CVID, chronic sinusitis, bronchitis, pneumonia, vitiligo, and congenital hypoplastic left kidney [13]. She had recurrent infections requiring multiple antibiotic treatments and testing at the department of immunology revealed that she had low levels of IgG and IgM and absent IgA and isohemagglutinins [13]. This confirmed her CVID diagnosis [13]. She was started on IVIg therapy [13]. She didn’t experience further bacterial infections but still suffered from bronchitis and had a significant increase in weight [13]. A CT scan showed mild bronchiectasis and interstitial lung inflammation [13]. She had difficult venous access, which was exacerbated with pregnancy and being that she was obese, this put her at increased risk for thromboembolic events [13]. During her pregnancy, she switched to SCIg treatment with the same dose and schedule as her IVIg treatment [13]. She had great success with this and was able to continue with home administration after discharge with administration training [13]. This case study showed that SCIg can be a viable option and have the same desired effects as IVIg [13].

Furthermore, previous data has suggested IVIg effects decrease in the second and third trimesters of pregnancy and SCIg can be an alternative [13]. Garduf et al. conducted a study that showed pregnant mothers with CVID who received SCIg delivered babies with adequate IgG levels [13]. However, in some cases, some patients may not want to make the switch to SCIg and think it is too tedious to undergo at-home treatments [13]. This was the case for a 36-year-old woman in a case study performed by Danieli et al. [13]. The patient had an unplanned pregnancy and felt the switch in treatment was too great of a burden [13]. It is important to educate pregnant patients with CVID on the different types of alternative therapies that can be easier and better for them and their babies in the long run. However, ultimately the patient’s autonomy must be respected and whichever treatment method he or she prefers is the most suitable.

In another case study by Horowitz et al., an 18-year-old male was observed [7]. He had an extensive history, including recurrent infections, Lyme disease, CVID, pediatric autoimmune neuropsychiatric disorders associated with streptococcal infections (PANDAS), and celiac disease [7]. He was also suffering from neuropsychiatric disorders, including attention-deficit and hyperactivity disorder (ADHD), likely attributable to heavy metal exposure at school [7]. He had low IgG levels, most likely due to concomitant Lyme disease, which is known to suppress humoral immunity [7]. He was also leukopenic, with a very low neutrophil count and low adrenal function, including low cortisol [7]. At age 18, he underwent human embryonic stem cell therapy (hSCT) [7]. This resulted in less frequent infections, immunoglobulin levels normalizing, decreased IVIg therapy, and resolution of leukopenia [7]. In addition, the symptoms associated with Lyme disease and neuropsychiatric improved [7]. His neck and back pain also decreased significantly [7]. This case study showed that specific disease processes can be exacerbated in CVID patients [7]. In this case, Lyme disease has been shown to affect IgG levels and make immunodeficiency more severe [7]. Heavy metal exposure, as was seen here, may also aggravate the immunodeficiency state [7]. According to a study by Wehr et al., there has been a variety of success rates for hSCT [7]. This may be due to the possibility of CVID having cell processes outside the hematopoietic stem cell line [7]. However, in this case, hSCT proved greatly successful in combination with continued IVIg therapy in a patient previously seeing little improvement on solely IVIg [7].

## Conclusions

CVID is an immune disorder, with limited awareness among practicing clinicians, especially PCPs. Since the condition mimics other immune diseases, it is frequently misdiagnosed or underdiagnosed, especially due to its varying disease presentation. There are no standardized criteria for diagnosis, with the mechanism of disease activity poorly understood. In most patients, environmental factors may contribute to the disease onset, however, in a smaller subset of patients, there are genetic factors involved. As a result, patients develop recurrent infections affecting multiple organ systems, especially sinopulmonary infections. Patients also have a poor immune response to vaccinations. The diagnosis of this condition is usually done through a process of elimination and treatment is based on presenting symptomatology and organ system affected. Physicians should consider all these factors when assessing patients with these clinical features, as diagnosing CVID patients earlier on can improve outcomes, decrease recurrent infections, and prevent the onset of serious co-morbid conditions.
